# Prolonged Opioid Usage After Surgically Treated Pelvic Fracture in Working Aged Patients: Prevalence, Demographics, and Multivariable Prediction Model

**DOI:** 10.1002/ejp.70247

**Published:** 2026-03-06

**Authors:** Elina Ekman, Rasmus Liukkonen, Aleksi Reito

**Affiliations:** ^1^ Department of Orthopaedics and Traumatology Turku University Hospital and the University of Turku Turku Finland; ^2^ Department of Orthopaedics and Traumatology Tampere University Hospital and the University of Tampere Tampere Finland

**Keywords:** epidemiology, Finland, opioid use, pelvic fractures, population‐based study, risk factors, socioeconomical status, working age

## Abstract

**Background:**

Pelvic fracture (PF) is a serious injury that can lead to prolonged opioid use. We examined opioid use after surgical treatment of PF in a working‐age population in Finland and identified predictors of prolonged opioid use.

**Methods:**

Using nationwide registries, we identified all Finnish inhabitants aged 18 to 65 years undergoing PF surgery between 2015 and 2021 (*n* = 233). These patients' demographic data, depression, trauma mechanisms, opioid purchases, and socioeconomic status were retrieved. The primary outcome was the prolonged opioid usage, defined as having more than one opioid prescription filled after the first three postoperative months. Logistic regression and Poisson zero‐inflated (ZIP) regression analyses were performed to examine the predictors of prolonged opioid usage. The results are reported as adjusted odds ratios (aORs) or adjusted incidence rate ratios (aIRRs) with 95% confidence intervals.

**Results:**

At 3–12 months and 6–12 months postoperatively, 40.3% and 33.0% of patients used opioids (at least one prescription), respectively. Preoperative opioid use (3–12 months: aOR 6.59, 95% CI [2.23, 24.38]; ZIP‐aOR 0.16, 95% CI [0.05, 0.50]; aIRR 2.19, 95% CI [1.81, 2.64]) was the single most important predictor of postoperative opioid use. Depression (aOR 2.08, 95% CI [1.09, 4.02]; ZIP‐aOR 0.48, 95% CI [0.25, 0.92]) showed modest effect at 3–12 months. Predictive performance of the regression for postoperative opioid use was low based on *R* ^2^ and AUC values.

**Conclusion:**

Prolonged opioid use was common. The predictive ability of the regression models for prolonged opioid use was modest and preoperative opioid use was the most important predictor.

**Significance Statement:**

In this nationwide registry study, we found that prolonged opioid use after surgically treated PF in Finland's working‐age population is common as 40.3% of the patients used opioids still over 3 months after the surgery. Preoperative opioid use and depression predicted prolonged opioid use. Also, preoperative opioid use, depression, younger age, PF operation during winter and falling or jumping from height were associated with increased opioid prescription volume. Opioids should not be prescribed after 3 months of the index surgery as one in seven PF patients became opioid dependent.

## 1 Introduction

Pelvic fractures (PFs), including pelvic ring and acetabular fractures, are relatively uncommon injuries. The reported incidence of PF in patients under 65 years ranges from 6.4 to 19 per 10^5^, with a male predominance (Verbeek et al. 2018; Rinne et al. 2020). PF in younger patients is often due to high‐energy trauma leading to severe injury that often requires surgical treatment (Hermans et al. 2017; Verbeek et al. 2018). A nationwide registry study reported that around 60% of PFs in patients under 60 years of age were treated operatively (Herteleer et al. 2021).

Given that PF is a serious injury—and often a high‐energy injury among young patients—functional impairment, chronic pain, and reduced quality of life are possible long‐term complications (Gabbe et al. 2015; Elgergawy et al. 2021; Banierink et al. 2022; Ng et al. 2023). In working‐age patients, this can lead to prolonged use of pain medication. In a recent meta‐analysis, opioid use more than 3 months after joint replacement surgery (JRS) of the hip, knee, or shoulder was reported in 12% of patients (Wu et al. 2021). According to data from the International Narcotics Control Board, global opioid use more than doubled in the first decade of the twenty‐first century (Berterame et al. 2016). As elective JRS aims to alleviate pain, discontinuation of opioids postoperatively should be given, especially when tackling an opioid crisis that we are faced with (Manchikanti et al. 2012). However, in cases of sudden trauma, opioid discontinuation may be more difficult. To the best of our knowledge, only two retrospective studies regarding opioid use after PF have been published, and both were based on the same patient group (Villa et al. 2023, 2024). No nationwide studies exist.

The main aim of this nationwide registry study is to report the demographics and prevalence of opioid use after surgically treated PF in a working‐age population in Finland. The secondary aim is to investigate how accurately baseline predictors can predict prolonged use. As the current recommendations for pain management advise opioids only for short‐term use (Joshi 2023), we hypothesised that opioid use would be minimal after the first three postoperative months.

## 2 Materials and Methods

This is a nationwide retrospective register study. Patient data were collected from the Finnish Care Register for Health Care (CRHC), the national register of Finland's Social Insurance Institution (SII) and Statistics Finland. The CRHC was founded in 1967, and all public and private hospitals in Finland are obligated to report patient data to the register. The register contains data on age, sex, domicile, external cause of injury, type of injury, primary and secondary diagnoses, type of hospital (public or private), duration of hospital stay, and operations performed during the hospital stay.

We searched the CRHC (www.thl.fi) for records with Nordic Classification of Surgical Procedures (NCSP) code NEJ50 (operation for fracture of pelvic ring), NEJ60 (operation for fracture of acetabulum) or NEJ70 (external fixation for fracture of pelvis) and any International Classification of Diseases (ICD‐10) diagnosis code indicating a fracture around the pelvis (S32.*) between January 1, 2015 and December 31, 2021. Only primary surgeries were included and reoperations were excluded. For each patient, only the first recorded surgical procedure for PF was included, excluding possible new PF record after the first one. For these patients, information on opioid purchases after and shortly before the surgical procedure for PF fixation was obtained from the SII (www.kela.fi). All prescriptions (whether made during the hospital stay or later) were included and only opioid prescriptions retrieved from the pharmacy were included. The in‐hospital opioid use is not recorded in the SII or CRHC and hence it was not included in the present study. Opioid purchases were divided into seven categories: 1–2 months preoperatively, 0–2 months preoperatively, 0–2 months postoperatively, 0–3 months postoperatively, 3–6 months postoperatively, 6–9 months postoperatively and 9–12 months postoperatively. To identify potential preoperative depression, information on N06 drug purchases (indicating depression) 12 months before PF fixation was obtained from the SII.

**FIGURE 1:**
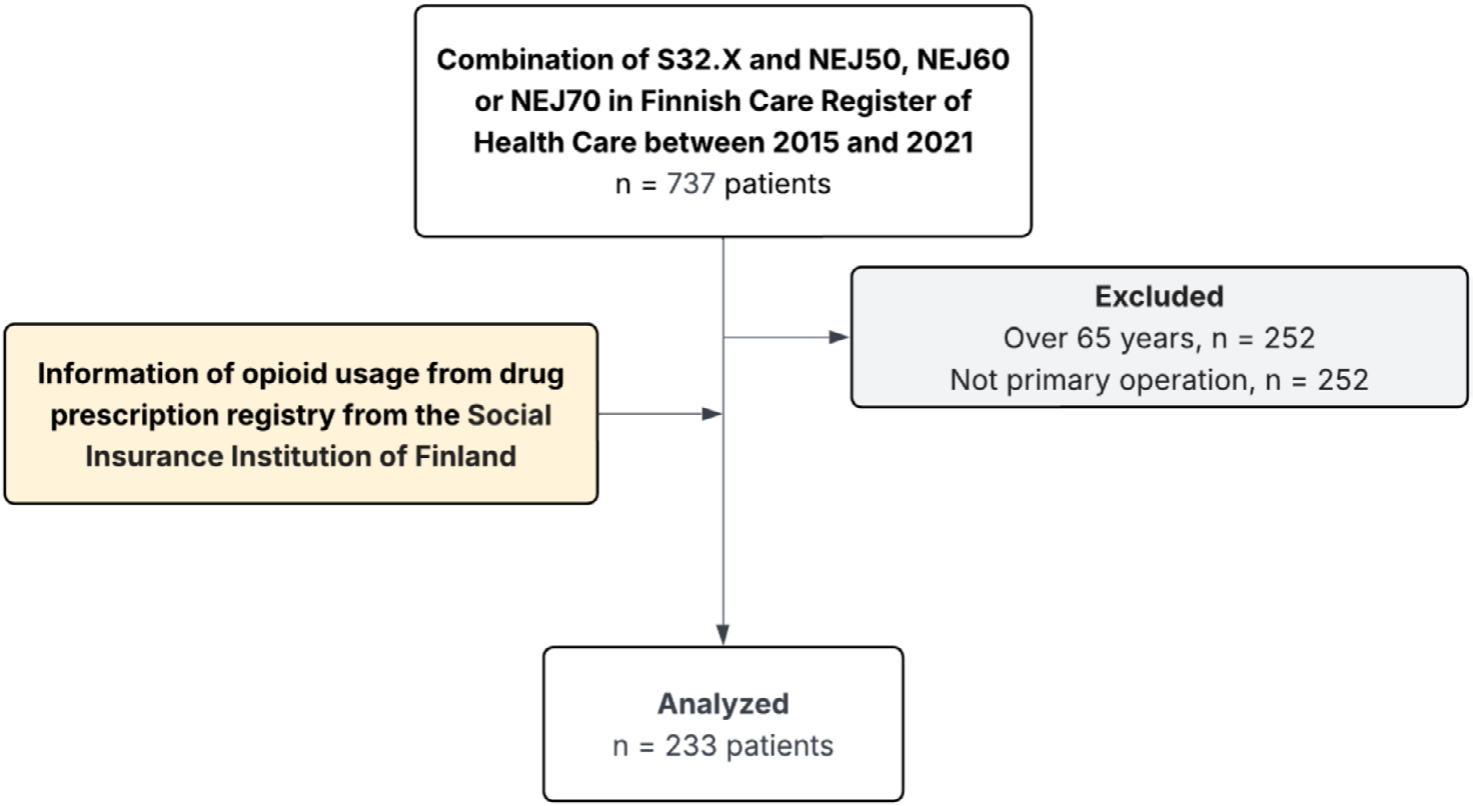
Inclusion flow chart.

Patients' socioeconomic status (SES) data were retrieved from Statistics Finland (www.stat.fi). SES was divided into four categories: (1) professionals and clerical staff, (2) manual workers, (3) entrepreneurs and non‐employed individuals and (4) unknown. Only working‐age inhabitants of Finland (18–65 years) were included in the study (see the inclusion flowchart in Figure 1).

The CRHC has exhibited substantial validity, especially in the case of orthopaedic trauma (Mattila et al. 2008; Sund 2012; Huttunen et al. 2014). The validity and completeness of Statistics Finland or SII data have not been studied. However, they both are government authorities and therefore considered valid. In Finland, opioids can only be bought from the pharmacy with a prescription, and SII is the only government authority to handle drug prescriptions. The completeness and validity of this data should therefore be 100%.

Ethical approval and the study permit were granted by Finland's National Institute for Health and Wellness (www.thl.fi, study permit number THL1785).

## 3 Statistics

Since we were only interested in opioid use, the Anatomical Therapeutic Chemical (ATC) codes used to identify pain medication prescriptions of interest were all oral and transdermal opioids (N02A***). The primary outcome was prolonged opioid use, defined as having one or more opioid prescriptions filled after the first three postoperative months (postoperative days 91–365). These definitions of prolonged opioid use and amount used were chosen since they are the most commonly used definitions in the related literature (Karmali et al. 2020). The secondary outcome was late prolonged opioid use, defined similarly but restricted to prescriptions filled after the first six postoperative months (postoperative days 181–365). All patients were followed for 12 months (365 days) after the index surgery; opioid prescriptions filled beyond this period were not included in the analyses. To complement these binary measures, opioid use was also evaluated as a continuous variable by recording the total number of prescriptions filled during the 12‐month follow‐up, as well as within the prespecified intervals of 0–3 months, 3–6 months, and 6–12 months. All analyses were conducted for the full study cohort, and no subgroup analyses were performed.

Logistic regression analyses were conducted to identify predictors of prolonged opioid use, and Poisson zero‐inflated regression analyses were conducted to identify predictors of zero use and the number of opioid prescriptions. Zero use was defined as no opioid prescriptions during the follow‐up period. The variables included in these analyses were based on previously known risk factors for prolonged opioid use. The zero‐inflated model consists of two components: a logistic component estimating the probability of having no opioid prescriptions during the follow‐up period (zero use), and a Poisson component estimating the number of opioid prescriptions among patients with at least one prescription. Covariates were included in both components of the model, and results were interpreted separately for predictors of zero use and for predictors of prescription count (Lambert 1992). All the results are reported as adjusted odds ratios (aORs) or adjusted incidence rate ratios (aIRRs) with 95% confidence intervals. The predictive ability of the regression models was examined with McFadden's *R* ^2^, and the discriminative abilities of the logistic regression analyses were examined with C‐indexes as expressed with the area under the curve (AUC). TRIPOD checklist was followed with regard to the clinical prediction model assessment. Internal validation was performed using bootstrap resampling (1000 repetitions). In each bootstrap sample, the full model including all prespecified predictors was refitted, and model performance was evaluated in the bootstrap sample and in the original dataset to estimate optimism. All the analyses were performed with *R*.

## 4 Results

**FIGURE 2:**
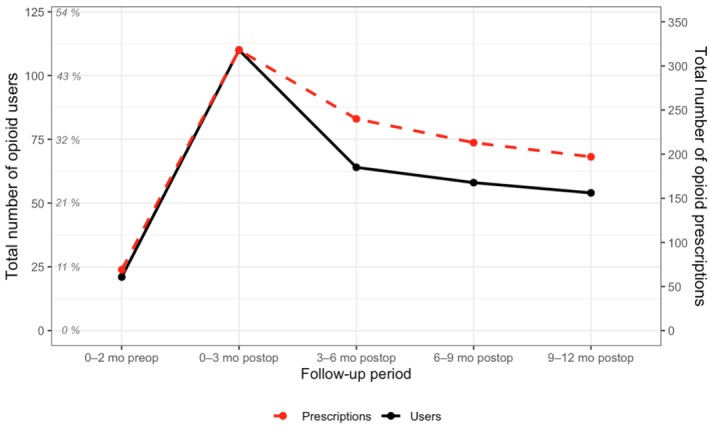
The numbers of opioid users and opioid prescriptions per follow‐up interval. The amount of opioid prescription filled at each timepoint for the entire study population. mo preop/postop = months preoperatively/postoperatively.

A total of 233 surgically treated PF patients were identified during the study period. The most frequent diagnosis code was S32.7 (“multiple fractures of the lumbar spine and/or pelvis”; 37.3%, *n* = 87). Patient demographics and fracture characteristics are detailed in Table 1. The number of patients who filled at least one opioid prescription and the number of opioid prescriptions filled during the study period are presented in Table 2 and Figure 2.

**TABLE 1:** Patient and fracture demographics.

	All patients (*n* = 233)
Male, *N* (%)	151 (61.8%)
Depression, *N* (%)	62 (26.6%)
Fracture operated during summer^a^, *N* (%)	119 (51.1%)
Age, mean (SD)	40.9 (14.7)
Age, median (IQR)	39 (28–55)
Time in the hospital, days, mean (SD)	17.1 (12.5)
Time in the hospital, days, median (IQR)	14 (9–21)
Trauma mechanism, *N* (%)	
Falling or jumping from height	76 (32.6)
Traffic accident	99 (42.5)
Other	58 (24.9)
Socioeconomical status, *N* (%)	
Professionals and clerical staff	43 (18.5)
Manual workers	40 (17.2)
Entrepreneurs and non‐employed individuals	104 (44.6)
Unknown	46 (19.7)

Abbreviation: IQR = interquartile range.

^a^ Time period between April 1 and September 30.

**TABLE 2:** The number of opioid users and the quantity of opioid prescriptions during the study period.

Timeframe	1–2 months pre‐op	0–2 months pre‐op	0–2 months post‐op	0–3 months post‐op	3–6 months post‐op	6–9 months post‐op	9–12 months post‐op	Total
Opioid users, *n* (%)	17 (7.3)	12 (5.2)	21 (9.0)	110 (47.2)	64 (27.5)	58 (24.9)	54 (23.2)	177 (76.0)
Opioid prescriptions, *n* (mean per patient)	38 (2.2)	31 (2.6)	69 (3.2)	318 (2.8)	240 (3.8)	213 (3.7)	197 (3.6)	1037 (5.9)

*Note:* The mean number of prescriptions is calculated for the patients with opioid prescriptions.

At 3–12 months postoperatively, 94 patients (40.3%) used opioids. Among the patients using opioids, the mean number of filled opioid prescriptions was 2.8 (SD = 7.0, range 0–71). At 6–12 months postoperatively, 77 patients (33.0%) used opioids, and the average number of filled opioid prescriptions was 1.8 (SD = 4.6, range 0–44).

Preoperative opioid use was a strong predictor of filling at least one opioid prescription postoperatively at both 3–12 months and 6–12 months (Tables 3a, 3b, 4a and 4b). Preoperative opioid use showed both the highest odds ratio and highest relative importance for prediction in relation to other predictors of postoperative use (Figures 1.1 and 1.2 in Supporting Information). Depression was associated with increased odds of filling at least one opioid prescription in the 3–12 months, but not the 6–12 months period (Tables 3a and 3b). Depression was also negatively associated with zero‐use in the Poisson model (Tables 4a and 4b). Younger age was significantly associated with an increased number of opioid purchases at both 3–12 months and 6–12 months (Tables 4a and 4b). Effects of other baseline predictors did not show consistent results in the analyses. Postoperative use of opioids stratified by preoperative use is shown in Table 5.

**TABLE 3a:** Opioid use 3–12 months postoperatively. Logistic regression analyses were used to identify predictors of prolonged opioid use.

Predictor	aOR	95% CI
Age	1.01	0.99–1.03
Female sex	1.56	0.85–2.87
Depression	2.08	1.09–4.02
Season (winter^a^ as reference)	0.89	0.50–1.56
Trauma mechanism (traffic accidents as reference)	—	—
Falling or jumping from height	0.73	0.36–1.44
Traffic accidents	1.00	—
Other	1.02	0.48–2.16
Length of hospital stay	1.00	0.97–1.02
Preoperative opioid use	6.59	2.23–24.38
Sosioeconomical status (manual workers as reference)	—	—
Professionals and clerical staff	0.74	0.29–1.88
Manual workers	1.0	—
Entrepreneurs and non‐employed individuals	0.50	0.22–1.14
Unknown	0.78	0.30–2.02

*Note:* McFadden *R* ^2^: 0.09 and AUC: 0.69.

Abbreviations: 95% CI = 95% confidence interval, aOR = adjusted odds ratio, AUC = area under the curve.

^a^ Time period between October 1 and March 31 (summer = April 1 to September 30).

**TABLE 3b:** Opioid use 6–12 months postoperatively. Logistic regression analyses were used to identify predictors of prolonged opioid use.

Predictor	aOR	95% CI
Age	1.02	1.00–1.04
Female sex	1.61	0.85–3.06
Depression	1.70	0.86–3.36
Season (winter^a^ as reference)	0.76	0.42–1.38
Trauma mechanism (traffic accidents as reference)	—	—
Falling or jumping from height	0.63	0.30–1.31
Traffic accidents	1.00	—
Other	0.95	0.42–2.08
Length of hospital stay	1.00	0.97–1.02
Preoperative opioid use	7.05	2.48–23.43
Sosioeconomical status (manual workers as reference)	—	—
Professionals and clerical staff	0.61	0.22–1.64
Manual workers	1.0	—
Entrepreneurs and non‐employed individuals	0.49	0.21–1.18
Unknown	1.11	0.42–2.97

*Note:* McFadden *R* ^2^: 0.10 and AUC: 0.69.

Abbreviations: 95% CI = 95% confidence interval, aOR = adjusted odds ratio, AUC = area under the curve.

^a^ Time period between October 1 and March 31 (summer = April 1 to September 30).

**TABLE 4a:** Opioid use 3–12 months postoperatively. Poisson zero‐inflated regression analyses were used to identify predictors of zero‐usage and the number of opioid prescriptions. Zero‐usage was defined as no opioid prescriptions during the follow‐up period.

Zero‐model	aOR	95% CI
Age	0.99	0.97–1.01
Female sex	0.63	0.34–1.17
Depression	0.48	0.25–0.92
Season (winter^a^ as reference)	1.09	0.61–1.92
Trauma mechanism (traffic accidents as reference)	—	—
Falling or jumping from height	1.31	0.65–2.65
Traffic accidents	1.00	—
Other	0.99	0.47–2.12
Length of hospital stay	1.00	0.98–1.03
Preoperative opioid use	0.16	0.05–0.50
Sosioeconomical status (manual workers as reference)	—	—
Professionals and clerical staff	1.40	0.55–3.61
Manual workers	1.00	—
Entrepreneurs and non‐employed individuals	1.99	0.86–4.61
Unknown	1.32	0.51–3.44

Count‐model	aIRR	95% CI
Age	0.99	0.98–0.99
Female sex	0.99	0.84–1.17
Depression	0.93	0.77–1.12
Season (winter^a^ as reference)	0.65	0.55–0.76
Trauma mechanism (traffic accidents as reference)	—	—
Falling or jumping from height	0.56	0.45–0.71
Traffic accidents	1.00	—
Other	1.11	0.90–1.37
Length of hospital stay	1.00	0.99–1.00
Preoperative opioid use	2.19	1.81–2.64
Sosioeconomical status (manual workers as reference)	—	—
Professionals and clerical staff	1.67	1.27–2.20
Manual workers	1.00	—
Entrepreneurs and non‐employed individuals	0.98	0.74–1.28
Unknown	1.54	1.17–2.03

*Note:* McFadden *R* ^2^ for count‐model: 0.14.

Abbreviations: 95% CI = 95% confidence interval, aIRR = adjusted incidence rate ratio, aOR = adjusted odds ratio.

^a^ Time period between October 1 and March 31 (summer = April 1 to September 30).

**TABLE 4b:** Opioid use 6–12 months postoperatively. Poisson zero‐inflated regression analyses were conducted to identify predictors of zero‐usage and the number of opioid prescriptions. Zero‐usage was defined as no opioid prescriptions during the follow‐up period.

Zero‐model	aOR	95% CI
Age	0.98	0.96–1.00
Female sex	0.60	0.31–1.16
Depression	0.58	0.29–1.16
Season (winter^a^ as reference)	1.27	0.69–2.33
Trauma mechanism (traffic accidents as reference)	—	—
Falling or jumping from height	1.45	0.68–3.12
Traffic accidents	1.00	—
Other	1.09	0.49–2.42
Length of hospital stay	1.00	0.98–1.03
Preoperative opioid use	0.15	0.05–0.46
Sosioeconomical status (manual workers as reference)	—	—
Professionals and clerical staff	1.68	0.61–4.61
Manual workers	1.00	—
Entrepreneurs and non‐employed individuals	1.97	0.81–4.77
Unknown	0.91	0.34–2.46

Count‐model	aIRR	95% CI
Age	0.98	0.97–0.99
Female sex	1.00	0.81–1.24
Depression	0.92	0.72–1.17
Season (winter^a^ as reference)	0.76	0.62–0.94
Trauma mechanism (traffic accidents as reference)	—	—
Falling or jumping from height	0.52	0.38–0.72
Traffic accidents	1.00	—
Other	1.19	0.91–1.54
Length of hospital stay	0.99	0.98–1.00
Preoperative opioid use	2.00	1.58–2.55
Sosioeconomical status (manual workers as reference)	—	—
Professionals and clerical staff	1.37	0.96–1.95
Manual workers	1.00	—
Entrepreneurs and non‐employed individuals	0.84	0.59–1.19
Unknown	1.27	0.90–1.79

*Note:* McFadden *R* ^2^ for count‐model: 0.13.

Abbreviations: 95% CI = 95% confidence interval, aIRR = adjusted incidence rate ratio, aOR = adjusted odds ratio.

^a^ Time period between October 1 and March 31 (summer = April 1 to September 30).

**TABLE 5:** The number of opioid users and opioid prescriptions postoperatively presented separately for opioid‐naive and non‐naive users. Opioid prescriptions are presented as absolute numbers during the whole study period and means and medians per patient.

Opioid prescriptions between 3 and 12 postoperative months	No preoperative opioid usage (*n* = 212)	Preoperative opioid usage (*n* = 21)
Number of opioid prescriptions, *N*	448	202
Number of opioid prescriptions, mean, SD	2.1 (6.1)	9.6 (10.5)
Number of opioid prescriptions, median, IQR	0 (0–1)	8 (2–12)

Opioid prescriptions between 6 and 12 postoperative months	No preoperative opioid usage (*n* = 212)	Preoperative opioid usage (*n* = 21)
Number of opioid prescriptions, *N*	275	135
Number of opioid prescriptions, mean, SD	1.3 (4.0)	6.4 (7.1)
Number of opioid prescriptions, median, IQR	0 (0–1)	5 (1–8)

Abbreviation: IQR = interquartile range.

The predictive ability of the regression models regarding prolonged opioid use was modest; for use after the first three postoperative months, the *R* ^2^ was 0.09 and the AUC was 0.69. After the first 6 months, these values were 0.10 and 0.69, respectively. The *R* ^2^ was 0.14 for the number of prescriptions at 3–12 months postoperatively and 0.13 for the number of prescriptions at 6–12 months postoperatively, indicating modest predictive ability. Results from the bootstrap internal validation, including apparent and optimism‐corrected performance metrics (AUC, calibration intercept, calibration slope, and Brier score) as well as calibration plots, are presented in the Supporting Information.

## 5 Discussion

PF requiring surgical treatment in the working‐age population is a serious injury. In our Finnish population‐based study, we found that opioid prescribing in this patient group was common, as 47.2% used opioids 3 months after the injury. Although opioid use declined over time, it remained notable—27.5% at 6 months and 23.2% at 12 months. Among those who used opioids at 1 year, 14.2% were opioid‐naive before the fracture. At 3–12 months postoperatively, 40.3% of patients used opioids, and at 6–12 months postoperatively, 33.0% used opioids. Additionally, the number of opioid prescriptions filled per patient rose between 0–3 months and 9–12 months postoperatively, indicating increased use among patients with prolonged opioid use.

To our knowledge, only two prior studies have examined opioid use following PF, both of which used the same U.S. patient cohort. These single‐centre retrospective studies (Villa et al. 2023, 2024) investigated adult patients treated for PF between 2015 and 2020. Whereas the 2024 study (Villa et al. 2024) focused solely on inpatient opioid use, Villa et al.'s (2023) 2023 study examined opioid use up to 60–90 days post‐discharge. Interestingly, the latter study reported that only 16% of patients used opioids during that period, which is substantially lower than the rate at 3 months identified in our study. However, when limiting the comparison to opioid‐naive patients at 3–6 months postoperatively, our findings approximate those of Villa et al.'s (2023) study, which also included non‐surgically treated patients (though the proportion was not specified). This may partly explain the lower opioid use rate observed in their study, as non‐operative cases are often less severe and involve less pain. Additionally, they found that PF type was an independent risk factor for prolonged opioid use. Although our study lacked fracture classification data, it included trauma mechanisms, but we found no association between trauma mechanisms and prolonged opioid use. Injuries caused by falling or jumping from height, however, were associated with an increase in the number of filled opioid prescriptions.

In line with our results, Villa et al. (2023) reported that neither age nor pre‐injury opioid use was associated with prolonged opioid use. In our cohort, age was only associated with the number of opioid prescriptions filled per patient, and pre‐injury opioid use correlated with filling at least one opioid prescription after surgery but not with continued use overall.

Although research on opioid use following PF is limited, Chaudhary et al. (2017) studied 13,624 patients following major trauma. The patients were aged 18–64 years with an Injury Severity Score (ISS) of ≥ 9. ISS is a numerical score ranging from 0 to 75, with higher scores indicating more severe injuries. The researchers found that although 53.9% filled at least one opioid prescription after discharge, use declined rapidly: 25.7% at 1 month, 8.9% at 3 months and 3.9% at 6 months. By contrast, our results suggest that Finnish patients with surgically treated PF use opioids for a significantly longer period, increasing the risk of dependence. However, Chaudhary et al.'s (2017) study included only patients insured by a single provider, so prescriptions paid for out‐of‐pocket were not captured, potentially leading to an underestimation of opioid use. Additionally, since an ISS of ≥ 9 includes moderate injuries, direct comparisons are limited. Also, a retrospective study on hip fracture patients found that depression was statistically significantly associated with increased perioperative opioid filling within 7 days pre‐operatively to 1 year post‐operatively (Cunningham et al. 2021). Although the patient material is somewhat different to the current study the effect of depression on opioid use suggests similar results.

Most of the surgically treated working‐age PF patients in our study were male. This aligns with Villa et al.'s (2023) findings, as they reported that 61% of their working‐age PF patients (aged 26–64) were male. Similarly, Verbeek et al. (2018), using data from the Dutch National Trauma Registry, found that 62.7% of PF patients under 65 were male. A single‐centre study conducted in Singapore (Singh et al. 2022) reported an even greater male predominance—79.3% of patients under 70. These findings are consistent with ours. The Singaporean study also reported that road traffic accidents (53.8%) and falls from height (28.4%) were the most common trauma mechanisms, which mirrors our results.

To date, no studies have investigated the association between SES and opioid use after PF. However, low SES—often defined as lower education level—has been associated with persistent opioid use 1–12 months after hip fracture, major trauma or total hip arthroplasty (Chaudhary et al. 2017; Kleno et al. 2024; Risbo et al. 2025). In our study, SES was not associated with opioid use after PF. This may reflect differences in patient cohorts, as our population included only working‐age fracture patients; thus, our results may not be directly comparable to those of the aforementioned studies.

We found that the predictive ability of the regression models for prolonged opioid use was modest. None of the included factors could predict prolonged opioid use, not even SES. Preoperative opioid use was also the strongest predictor of purchasing opioids postoperatively. Also, the number of opioid prescriptions filled per patient was higher in patients who used opioids preoperatively compared to opioid‐naive patients. It seems that predicting who will become opioid dependent after major trauma is difficult. Focusing on individual predictive factors might also be fruitless without an overall model. The easiest way to avoid patients becoming addicted to opioids is not to prescribe them beyond the initial phase.

This study has some limitations. First, because it relied on registry data, detailed fracture classifications were unavailable. The CRHC contains only ICD‐10 codes, which limited our ability to compare findings with fracture classifications used in other studies. Second, the quality of registry data depends on accurate reporting. SES data were frequently missing, which may have reduced the statistical power needed to detect a significant association. Also, missing information can introduce selection bias if not all patients are recognised from the registry. The validity of CRHC is reported to be good; we believe that the risk for bias is minimum. However, the validity of CRHC regarding reoperations has not been studied; hence it might be possible to confuse reoperations with a second operation due to another PF during our study period. To avoid overestimation, we chose only to include the first recorded surgical procedure for PF. This choice, of course, can slightly underestimate the number of PFs, but we believe this to be a minor bias. Third, information on previous pain conditions (such as chronic pain syndrome), mental health problems (other than depression), and substance misuse and the use of medication for chronic pain were not available for this study, and their influence on patient recovery and opioid use could not be estimated. This can lead to confounding bias. This bias might be significant in patients with falling or jumping from height as their trauma mechanism because this trauma mechanism can include pronounced amounts of patients with mental health problems or substance misuse. These patient‐related factors might explain the increased number of opioid purchases related to this trauma mechanism. Forth, it is possible that the opioids are prescribed for other conditions than PF, overestimating our results. Again, we feel this risk is small due to the small likelihood of patients suffering some other trauma while recovering from PF. Also, the current study includes only surgically treated PFs of the working‐age populations, limiting the generalisability of our results outside this population. Hence, the results only apply to the most severe and surgically treated PF patients accounting for 60% of all PF patients.

A key strength of our study is its use of nationwide data rather than data from a single hospital or insurance provider. Moreover, the validity of the CRHC has been shown to be high, particularly in orthopaedic trauma (Mattila et al. 2008; Sund 2012; Huttunen et al. 2014), supporting the reliability and generalisability of our findings. Also, the validity of external cause of injury and type of injury in the CRHC has increased during the study period as the coding for external cause of injury and type of injury has increased and surpassed 90% by the year 2010 due to the advancement of the electronic patient record system (Hallinen et al. 2025). In the current study, either external cause of injury or type of injury could be found for each patient.

At 1 year post‐injury, 23.2% of patients were still using opioids, 14.2% of whom were new users. This means that one in seven previously opioid‐naive PF patients at 1 year post‐injury was dependent on opioids. This result is alarming—especially given the lack of clear evidence for the long‐term effectiveness of opioids in managing chronic pain (Rosenblum et al. 2008). We also found that, instead of declining, the quantity of opioids used per patient 12 months after PF surgery increased and that younger patients tended to use larger quantities of opioids than older patients. Physicians in Finland should be especially cautious when prescribing opioids beyond 3 months after PF surgery. Further research is needed to identify the root causes of opioid dependency. Are patients left alone with their problems after major traumas like PF? Could systematically providing psychological support help prevent prolonged opioid use?

## Author Contributions

This study was designed by A.R., E.E., and R.L. E.E. drafted the manuscript. R.L. calculated the statistics. E.E., R.L., and A.R. contributed to the interpretation of the data and results and to the preparation of the manuscript. All authors read and approved the final manuscript and agree to be accountable for all aspects of the work.

## Funding

This study received funding from the Research Council of Finland. The funder had no involvement in the study.

## Conflicts of Interest

The authors declare no conflicts of interest.

## Supporting information


**Data S1:** ejp70247‐sup‐0001‐DataS1.docx.

**Data S2:** ejp70247‐sup‐0002‐DataS2.docx.

## Data Availability

The data supporting the findings of this study are available from the corresponding author upon reasonable request.
