# Supplementary material for: Prolonged Opioid Usage After Surgically Treated Pelvic Fracture in Working Aged Patients: Prevalence, Demographics, and Multivariable Prediction Model
Source: Eur J Pain. 2026 Mar 6;30(3):e70247. doi: 10.1002/ejp.70247 (PMC12966626; doi:10.1002/ejp.70247)
Supplement: Supplementary file 1 — Data S1: ejp70247‐sup‐0001‐DataS1.docx. [file EJP-30-0-s002.docx]

**Supplementary figure 1.1**: Variable importance from logistic model estimating opioid-use at 3-12 months.


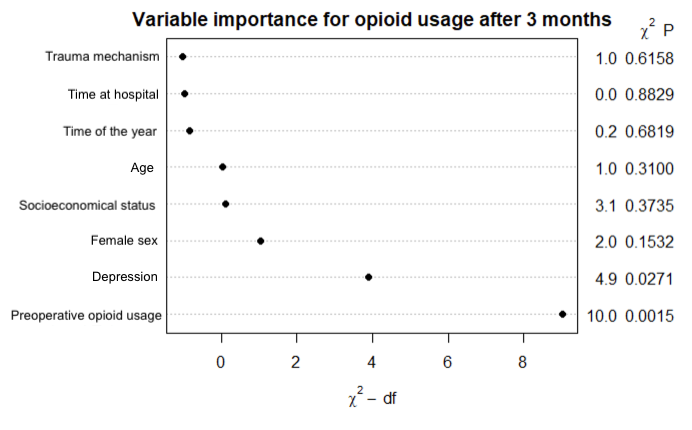


**Supplement figure 1.2**: Variable importance from logistic model estimating opioid-use at 6-12 months.


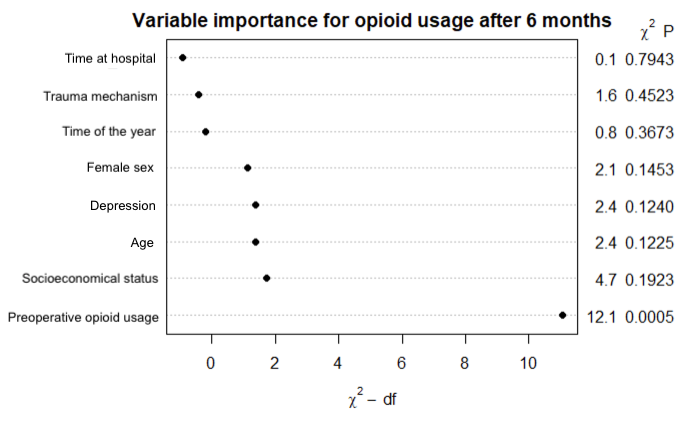


|  | **Usage after 3 months** | | **Usage after 6 months** | |
| --- | --- | --- | --- | --- |
| **Metric** | **Apparent** | **Optimism-corrected** | **Apparent** | **Optimism-corrected** |
| AUC | 0.69 | 0.62 | 0.69 | 0.62 |
| Calibration intercept | 0.00 | -0.14 | 0.00 | −0.21 |
| Calibration slope | 1.00 | 0.67 | 1.00 | 0.69 |
| Brier score | 0.21 | 0.24 | 0.19 | 0.21 |

**Supplementary table 1.** Apparent and optimism-corrected performance of the logistic regression models predicting postoperative opioid use.

|  |  | **Usage after 3 months** | **Usage after 6 months** |
| --- | --- | --- | --- |
| **Predictor** | **Coding** | **β coefficient** | **β coefficient** |
| Intercept | — | −0.7469 | −1.1940 |
| Age | Per 1-year increase | 0.0105 | 0.0169 |
| Sex | Male vs female (reference) | 0.4430 | 0.4750 |
| Socioeconomical status | Professionals & clerical staff vs manual workers (reference) | −0.3035 | −0.4952 |
| Socioeconomical status | Entrepreneurs and non-employed vs reference | −0.6926 | −0.7091 |
| Socioeconomical status | Unknown vs reference | −0.2454 | 0.1057 |
| Depression | Yes vs no (reference) | 0.7336 | 0.5326 |
| Preoperative opioid usage | Yes vs no (reference) | 1.8862 | 1.9536 |
| Length of hospital stay | Per 1 day increase | −0.0017 | −0.0032 |
| Cause of injury | Other vs traffic accident (reference) | 0.0185 | −0.0547 |
| Cause of injury | Fall vs reference | −0.3194 | −0.4616 |
| Season | Non-summer vs summer (reference) | −0.1179 | −0.2731 |

**Supplementary table 2.** Full specification of the logistic regression model predicting opioid use after surgery.


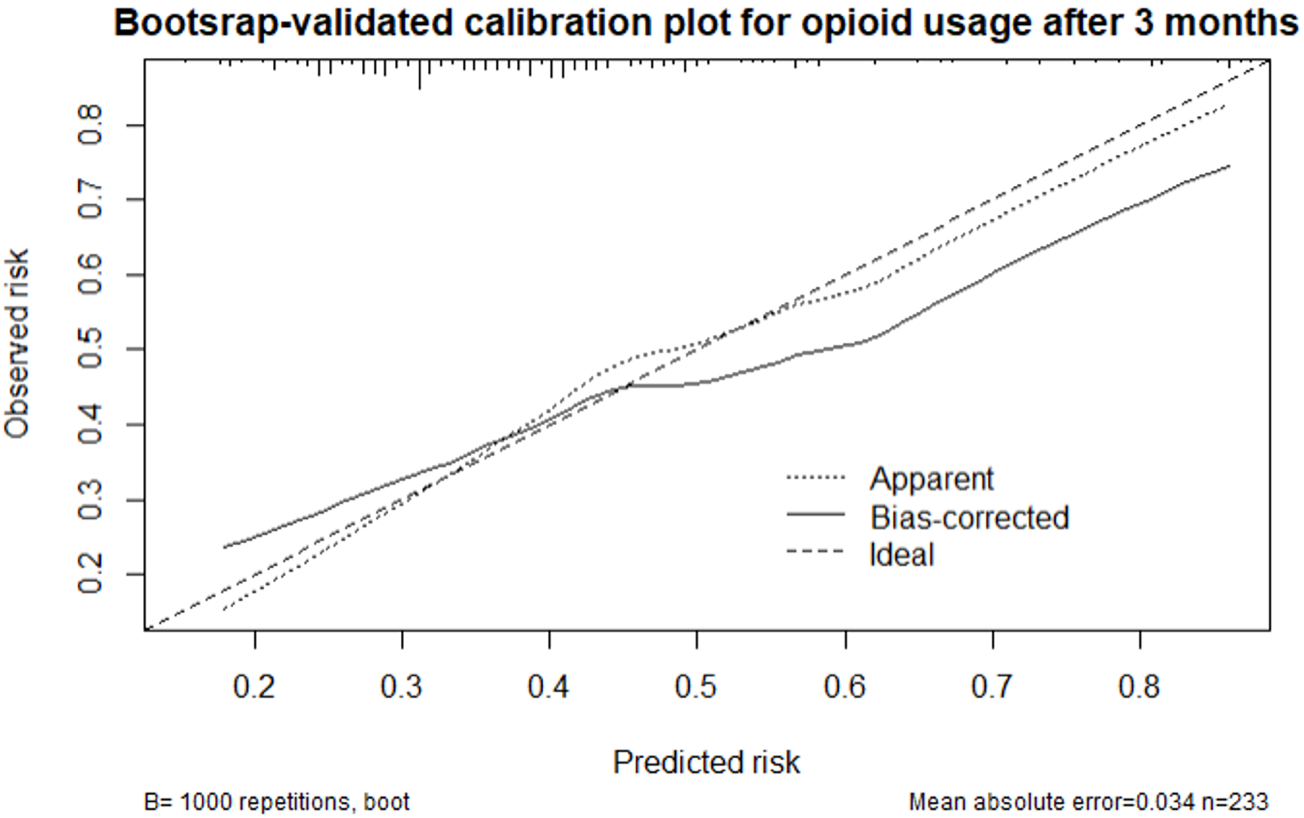


**Supplementary figure 1.** Bootstrap-validated calibration plot for the logistic regression model predicting opioid use 3 to 12 months after surgery. The solid line represents optimism-corrected calibration; the dashed line indicates ideal calibration.


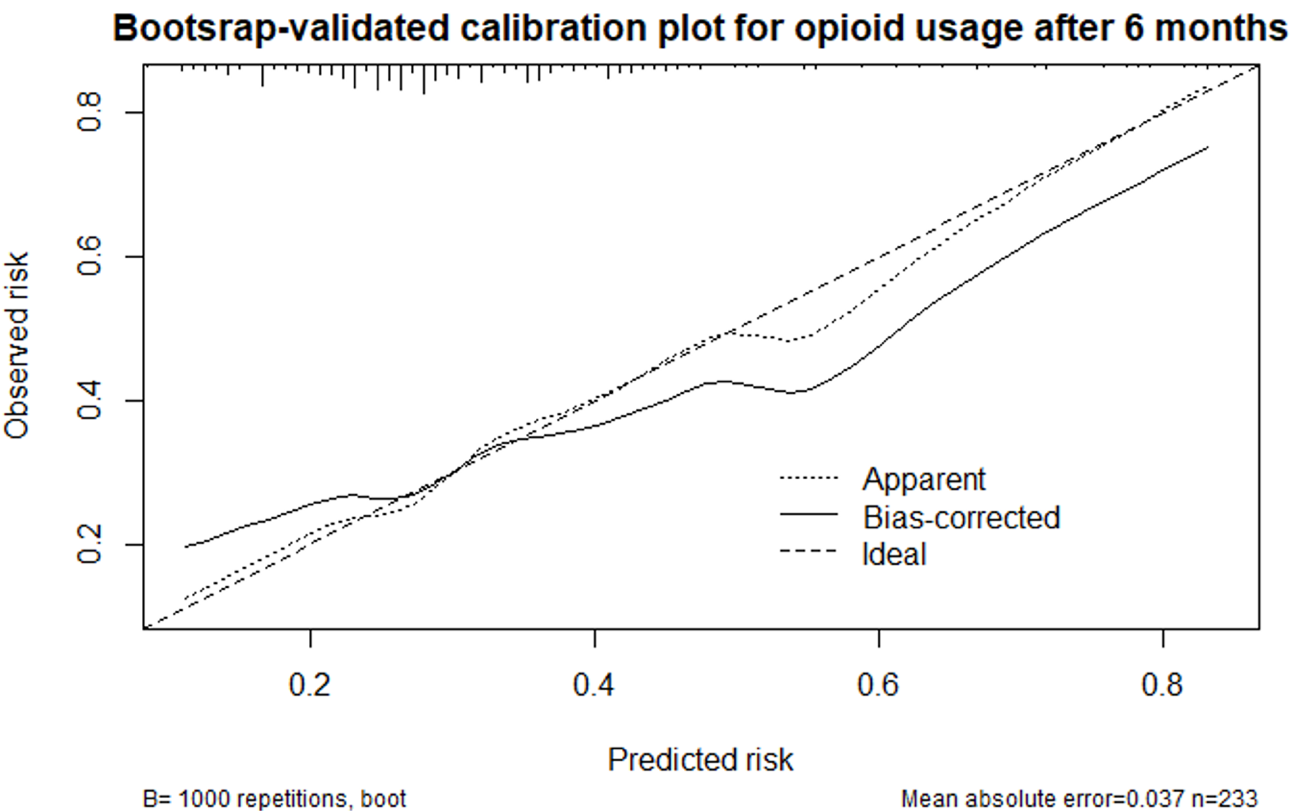


**Supplementary figure 2.** Bootstrap-validated calibration plot for the logistic regression model predicting opioid use 6 to 12 months after surgery. The solid line represents optimism-corrected calibration; the dashed line indicates ideal calibration.
